# Augmented Reality (AR) Supporting Citizen Engagement in Circular Economy

**DOI:** 10.1007/s43615-021-00137-7

**Published:** 2022-02-03

**Authors:** Tina Katika, Ioannis Karaseitanidis, Dimitra Tsiakou, Christos Makropoulos, Angelos Amditis

**Affiliations:** 1 Institute of Communication and Computer Systems of the School of Electrical and Computer Engineering, Athens, Greece; 2 Department of Water Resources and Environmental Engineering, School of Civil Engineering, National Technical University of Athens, Athens, Greece

**Keywords:** Augmented reality, Citizen engagement, Circular economy, Web survey, User engagement

## Abstract

Improving the public’s awareness of and engagement with sustainable practices is essential for transitioning from a linear to a circular economy (CE). This task is not trivial; however, research into new ways of effectively supporting awareness and engagement, especially in view of digitalization trends, is still missing. In this study, we investigate the ability of augmented reality (AR) technology to improve awareness of CE practices and to engage citizens, inclusively, in the CE approach. Specifically, we report on the development of a novel AR engagement tool and present the results of two studies undertaken using this tool to assess the reliability and validity of this engagement practice. The first study focuses on the citizens of a municipality in Greece, and the second focuses on the general public outside the municipality boundaries. Attributes related to user engagement, such as novelty, aesthetics, perceived usability, endurability, focused attention, self-efficacy, perceived learning, and interest in the topic, were assessed, as were factors affecting these attributes related to the participants’ knowledge of CE principles and exposure to similar technologies. The two studies indicate that the AR engagement tool increased social inclusion and community cohesion and was able to successfully bring CE principles and benefits to the public’s attention. Higher engagement levels were observed in participants who had limited exposure to the concept of CE prior to using the AR tool, and its use was found to improve their confidence and interest in CE. This study aspires to contribute to the ongoing discourse on the use of mobile AR and to support further evidence-based development of digital tools for public awareness and engagement.

**Supplementary Information:**

The online version contains supplementary material available at 10.1007/s43615-021-00137-7.

## Introduction

It is understood that to progress towards sustainable living effectively, citizens in all their diversity must be tightly engaged [, 1, 2]. Towards this goal, a transition to a circular economy (CE) model is needed. Such a transition needs to be supported by behavioral, social, and cultural changes, as well as new partnerships between governments and citizens, private and public sectors. Citizen engagement can only be fostered in the context of productive dialogues that reflect the needs of a municipality [3] and where citizens are able to weigh a variety of ideas and build a shared understanding of potential choices as well as emerging opportunities and threats [4]. Such a means draw citizens into a social process, sharing knowledge and generating ideas, hence directly countering exclusion and isolation, provided that the citizen groups are inclusive (i.e., not biased by gender, social differences, or other heterogeneities). In this regard, the need for using a common language to explain and communicate global issues and thereby involve citizens across scales, backgrounds, and identity boundaries is being acknowledged [5].

An effective citizen engagement process should include the voices and needs of all participants and increase their knowledge about a public issue, encouraging them to apply that knowledge and finally what they learned to improve their quality of life and the community [4, 6]. Creating opportunities for citizens to engage each other, and ensuring that these opportunities are regular and ongoing, contributes to the long-run success of these initiatives.

New technologies and digital transformation play an essential role in the process of citizen engagement and active participation, helping to reconfigure social relations and empower citizens, connect individuals, and facilitate knowledge exchange across ever-widening specificities [7]. Several engagement tools have thus been developed to engage people in their physical communities and enable participation [4]. Such initiatives have demonstrated that empowered and committed citizens transform from passive audiences to interactors, and immersive technologies and interactive media are well placed to generate such transformative experiences [4].

Over the past decade, media, technologies, software, and cultural practices that change how we experience and interact with the environment have emerged. Various Information and Communication Technologies (ICT) tools, including websites, mobile solutions, social media, and platforms, have been proposed to improve the understanding of public matters and engage citizens in a great variety of topics [4]. Nevertheless, despite these efforts, such initiatives often fail since they are not inclusive [4, 6] or they are designed for a specific target group (such as expert users and stakeholders), or they tackle appropriately either young or senior audiences. Their content is often limited, not engaging enough, and the engagement tools appear restrictive in terms of portability, usability, and accessibility; or they do not comply with a specific framework or measures [4, 6]. A large-scale web-based citizen participatory approach has been demonstrated by Stern et al. [8]. The authors revealed a significant bias of the web-based public participation processes regarding participants of different age groups and educational levels. The public participation processes appeared exclusive at the educational level, as they only reached 14.3% of the participants without higher education. Significant differences were also observed in the age of the participants, as the most significant percentage demonstrating engagement was between 29 and 39 years of age. Finally, the female population engaging in the web-based participatory approach was twice the size of the male one [8].

Regarding the overall usability of ICT-based engagement practices for societal issues, Mukhtarov et al. [9] provided a systematic review in 32 case studies to study public participation in urban water governance. The authors explored crowdsourcing and platform-based solutions, as well as social media, open-source software, blogs, and virtual learning platforms, and reported improvements in effectiveness and efficiency in urban water governance [9]. All engagement efforts demonstrated the essential components of an engagement tool to communicate, educate, and change the behavior of citizens towards environmental issues, but further investigation in terms of inclusivity or other attributes was not performed [9]. Church [6] demonstrated the usefulness of methodologies encouraging participation from marginalized groups. An effective ICT solution should emphasize the inclusivity of the citizens, where past experiences, lack of knowledge, and cultural context do not limit involvement. Sections of the community that have not participated in the past and might not seem fertile ground for recruitment should be reached. An impactful engagement process is built upon diversity and equality. All members of a community need to participate in representing different viewpoints and interests, and it should be clear that everyone respectfully participates on an equal basis. The work of the community group needs to be open, transparent, and consistent. At the same time, the engagement process should create opportunities for learning and use or applying that knowledge further at a non-restrictive timeframe and pace [4]. It is, therefore, apparent that engagement tools should be inclusive, accessible, and transparent and support learning and long-run well-being to engage all citizens with their heterogeneities.

Europe’s communities are in many ways uniquely placed to transition to a CE model that aims to improve citizen’s life quality in many environmental, social, and economic aspects, and citizens — of all ages and skills — need to be positioned in the center of this process [10]. Nevertheless, the lack of familiarity and fear of the unknown slows down the traction that the CE idea should have already gained [10]. Enhancing social responsibility and learning should improve public participation. Being able to include a wide range of different interests and concerns in a decision-making process, we will have greater chances of achieving acceptance of environmental and social decisions [11]. Despite this belief, enabling citizens to participate in public actions is, up until today, a significant challenge to solve social and environmental issues [12]. Being an open challenge since the Rio Declaration in 1992 [13], public environmental policies can only be relevant and functional if they involve all the citizens concerned. Therefore, it is crucial to make the intention and message clear for changing to a CE model and at the same time create a welcoming atmosphere for all citizens to participate.

Given the immersive nature of AR technology, the extended use of smartphones and the internet on the go, and the ability to couple them together with advanced location and camera settings, AR can be part of the citizen participatory process. AR has been reported to be achieving a turning point for large-scale adoption, while vast expansion is expected in the upcoming years [14]. Having the ability to fundamentally alter how we interact with content, allowing end-users to feel closer to global issues, enabling a form of telepresence that evokes empathy levels as if one were present, AR’s immersive nature helps the audience see details, believe in actions, and make connections between the events in the story and their own lives [15]. Consequently, end-users are able to understand the positive impact of specific policies and changes [16]. Digital media are conveyed interactively, and physical experiences are recreated and enhanced with virtual content that enables participants to move beyond static images and gives them the freedom to choose any viewpoint and explore [17]. Mobile AR offers the advantage of portability, mobility to the end-user, being accessible and available [18].

Given the adaptable nature of the technology, mobile AR can comfort the limitations that other engagement tools face. Immersive technologies have demonstrated a significant effect in educating end-users [17] and offer an inclusive environment for people having a wide range of specificities [19]. Up to our knowledge, a similar solution has not been developed to leverage advanced mobile functionalities, gamification, and educational features for CE training purposes. Small-scale projects have been proposed, but they have been examined in a narrow target group or fail to extract concrete results due to the lack of adherence to specific research frameworks or assessment models (e.g., [20–22].

In the present study, we contribute further to the discourse of citizen engagement tools by developing and exploiting a user-friendly mobile application to engage citizens in CE principles and empower a sense of action towards this change. Our motivation is to effectively apply an AR-based tool to motivate and educate citizens around the notions of a CE model. Using an AR application to foster engagement, we exposed a municipality’s citizens to digital content related to a CE approach. A web survey was utilized to recruit the citizens and collect the required data. We recruited 127 citizens of a municipality in Greece to investigate which attributes and factors affect their engagement and self-efficacy. Concurrently, we collected data from a second sample (of 101 participants) outside this municipality to investigate the validity of our questions and extract preliminary results to an extent more significant than that of one municipality. This paper presents the results of this study and tries to draw some conclusions in the primary research hypothesis that the use of AR can prove a valuable tool for a mental shift towards CE approaches at a citizen level in an inclusive, accessible, and educative manner.

The paper is organized as follows: in the following section, we discuss research on user engagement and explore the attributes and factors associated with engagement. We proceed by describing our research methodology and substantiating our analytical approach. The AR system is described then to demonstrate the features that affected engagement and assist future development efforts. We continue in our final section with a report of the results from the two research studies and conclude with a discussion section of how our findings relate to the improvement in citizen engagement and self-efficacy in CE, while we describe our limitations and provide suggestions for future research.

## User Engagement

User engagement is the emotional, cognitive, and behavioral connection that exists, at any point in time and possibly over time, between a user and a resource [23]. User engagement with a technological resource is not just about how a single interaction unfolds but about how and why people develop a relationship with technology and integrate it into their lives. Within this context, the quality of the user experience drives the positive aspects of the interaction, particularly the phenomena associated with being captivated by technology [23]. In human–computer interactions, engagement describes the combination of the user’s expectations and motivation, the purpose and functionality of the designed system, and the environment where the interaction occurs. Such experiences address the users’ need for being stimulated, perfect their skills and knowledge, and finally help them grow. Thus, user engagement is conceptually a holistic framework for understanding user and system variables’ integration and how they combine to push the boundaries of user experience from merely perfunctory to pleasurable and memorable [24]. Therefore, engagement builds upon the foundation of a usable system that is effective, efficient, and satisfactory. Following, we discuss some characteristics, attributes, and measures which are associated with user engagement.

## Attributes Affecting Engagement

The engagement process involves several stages, starting from initiation, continuing with a period of engagement, then disengagement, and reengagement. Several attributes are present in each stage and define the engagement process [24]. The engagement process initiates when the user’s aesthetic resonates with the system interface’s informational composition. Then, the participants’ attention marks the period of sustained engagement, and interest is maintained in the interaction either by presenting feedback or novel information and features on the interface. If disengagement occurs, the users re-engage when having fun, being rewarded with convenience and incentives, and learning or discovering something new. The presence of visual, auditory, and tactile elements in the interfaces further engages the user’s senses during their interactions [24].

One of the attributes affecting user engagement is the aesthetic appeal, which refers to the app’s visual elements. An aesthetically appealing ICT tool gives more pleasure and enjoyment, leading to a higher probability of using it. A significant component contributing to a mobile app’s aesthetic appeal is the user interface (UI), which refers to the flow of experience when users navigate an app, and whether performing tasks is user-friendly and convenient. According to Tang [25], it is essential to have clear and understandable instructions to give end-users a good experience flow. Focused attention is an engagement attribute that is often used to define the user’s motivation and reflects the interest towards the activity performed [26].

Focusing on AR tools, attention improves when the AR content is multimodal, and users can interact. The immersive capabilities of AR help users maintain high levels of attention and interest in the learning content, positively impacting users’ attention and engagement [26].

Novelty reflects the software or tool’s view as new, attractive, and identifiably different from others used or understood at the time of the introduction [27]. Novelty plays a meaningful role in users’ evaluation of visualized content. It can inspire interest, leading users to seek further information about a specific topic (in our case, the CE model) [27]. Novelty has been found to improve enjoyment, which is a crucial factor in influencing engagement [28]. Perceived usability refers to a user’s ability to perform tasks safely, effectively, and efficiently while enjoying the experience. It also predicts users’ lasting impressions of the experience and their willingness to engage with the application at another point in time [28]. Poor usability is a barrier to engagement and, therefore, is often connected to the ease of use and measures the ease users can experience while trying to complete the desired task with a product [24]. The aesthetics and novelty of AR appear to influence usability, which offers a significant value to the overall engagement. Felt involvement describes how much fun users have during the interaction and how drawn they are during their experience. If the usability of a system does not prevent the users from enjoying themselves, then the felt involvement is high, according to O’Brien and Toms [24]. Endurability describes the desire to redo an activity that has been fun or recommend it to others [24]. Endurability should sum up how rewarding, successful, and worthwhile the interaction with the AR app is. It also corroborates the influence of user and system variables on overall perceptions of experience.

Other attributes affecting user engagement are often associated with the interest, value, affect, and conscious awareness of motivation towards a particular activity or situation [29]. Individuals willing to approach a task in pursuit of a specific goal can be described as exhibiting motivational engagement. Motivational engagement is a prerequisite for productive learning as it is associated with achievement, motivation, task persistence, and meaningful processing on achievement measures [30]. Users of technologies that experience high levels of self-efficacy result in enhanced motivational engagement [29]. Self-efficacy is a motivational construct that is key to promoting users’ engagement and learning and refers to the beliefs about the capabilities one has to perform a specific task. It also involves some judgment that the user can or cannot do an activity. Finally, personal interest in the task and the content itself results in higher learning and comprehension and, therefore, engagement [31].

In our approach, and following the aforementioned analysis, we posit a model that examines nine attributes affecting user engagement, notably (i) perceived usability, (ii) felt involvement, (iii) focused attention, (iv) novelty, (v) aesthetics, (vi) endurability, (vii) interest in the topic of CE, (viii) perceived learning, and (ix) self-efficacy. The first six attributes affect user experiences with technology and define user’s intentions. The last three attributes affect the user’s motivation towards a specific topic or content (in our case, CE). The model’s reliability and validity are evaluated in the context of citizen engagement in CE in the following sections.

## Methodology

### Measurement and Research Questions

The User Engagement Scale (UES) was introduced by O’Brien and Toms [24], as means of measuring engagement, improving understanding of the user experience, and evaluating software solutions. The UES measures the self-reported user engagement based on a variety of attributes, such as focused attention, perceived usability, the attractiveness and visual appeal of the interface, users’ willingness to recommend an application to others or engage with it in future, novelty, and felt involvement [32, 33]. The UES has been proposed and validated to assess user engagement, guide digital media design, or evaluate user experience with computer-mediated systems. The suitability of UES in various domains has been demonstrated, and the developed UES survey is suitable to assess a great range of goals, ranging from engagement to tasks that allow the user to pass their time to an in-depth understanding of current issues [34]. In the present study, we further adopted the proposed scale to assess user engagement in the AR tool developed to educate and engage in CE matters. To develop the engagement tool, we take advantage of AR’s capabilities, such as object manipulation, outdoor localization, vivid digital content, a virtual assistant, and other gamification and educational features, to positively affect citizen engagement related to CE and foster an inclusive and accessible learning environment.

To address the limitations of previous efforts, we built an AR engagement tool aiming to improve social cohesion by considering factors such as age, disability, low income, low education, cultural or language differences, and geographical or social isolation [4]. A guiding factor of our research is also to determine an inclusive environment for educating citizens in complex and novel concepts that they have not been exposed to in the past. The qualitative study of citizen engagement used across these contexts was guided by the following three questions:

1. Is citizen engagement through AR technology inclusive for participants with low CE literacy and confidence on this topic?
2. Is citizen engagement through AR technology inclusive for participants of all age groups, educational levels, and genders?
3. Is citizen engagement through AR technology inclusive for participants who have never been exposed to similar technologies?

Answers to these questions are important as more extensive knowledge concerning citizen engagement may assist in designing inclusive and adaptive engagement tools with educative character and ultimately impact the adoption of CE principles. We also sought to provide insights that would help transform citizens of all ages from passive audiences to interactors regardless of their tech-savviness and prior exposure to technology.

In our study, we exposed 127 citizens of a municipality and 101 citizens outside this municipality to the AR engagement tool and investigated at first the impact of specific attributes on user engagement and, ultimately, how specific factors affected them. The nine attributes that affect citizens’ engagement, as described above, and three factors affecting these attributes (demographics, tech-savviness, and CE literacy, and confidence) were assessed.

### Development of the AR App

**Fig. 1:**
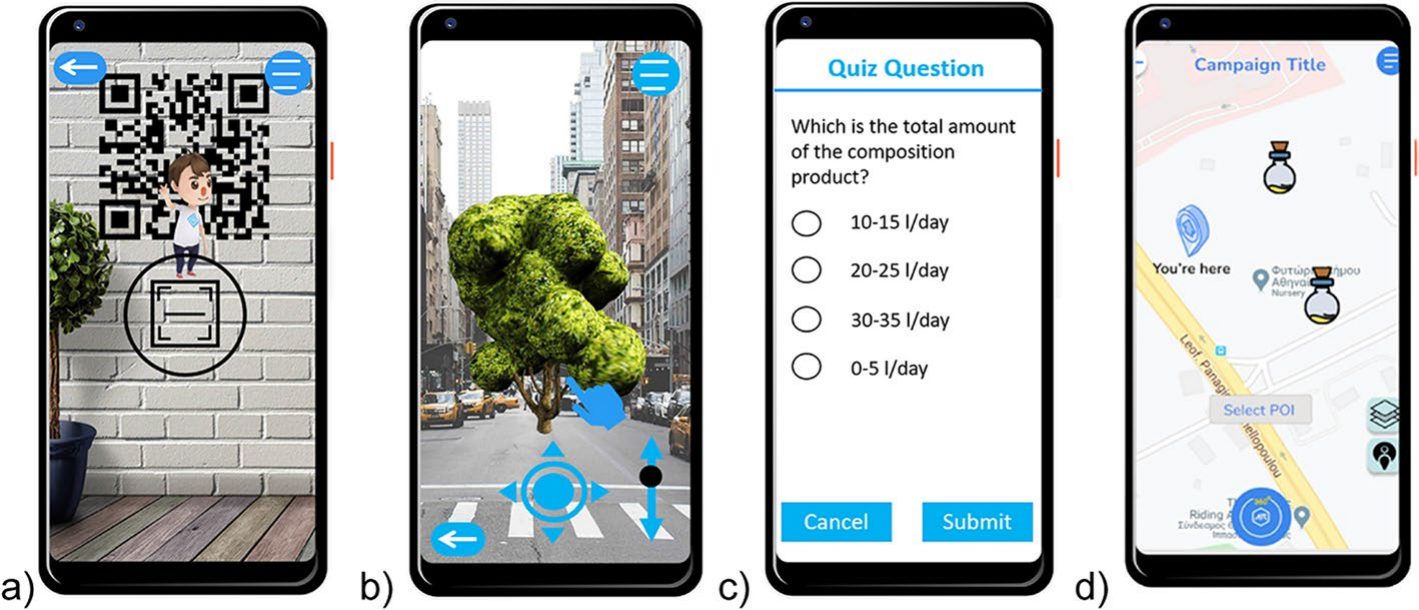
The user interface (UI) of the AR engaging tool with an open camera setting, showing the virtual assistant. The user is prompted to scan the QR code and view the digital media. b) A UI with an open camera setting, demonstrating CE-related content to the user. The media is activated based on the GPS location of the user. The hand indicates that the user is able to rotate the 3D virtual tree that overlays the physical surroundings. The arrows and cursor indicate the ability to move the virtual object around the physical surroundings. c) A UI with a quiz question that enables the user to test and validate their knowledge and understanding based on the virtual content they were previously exposed. d) The map with instructions navigating the user to the nearest AR experience

**Fig. 2:**
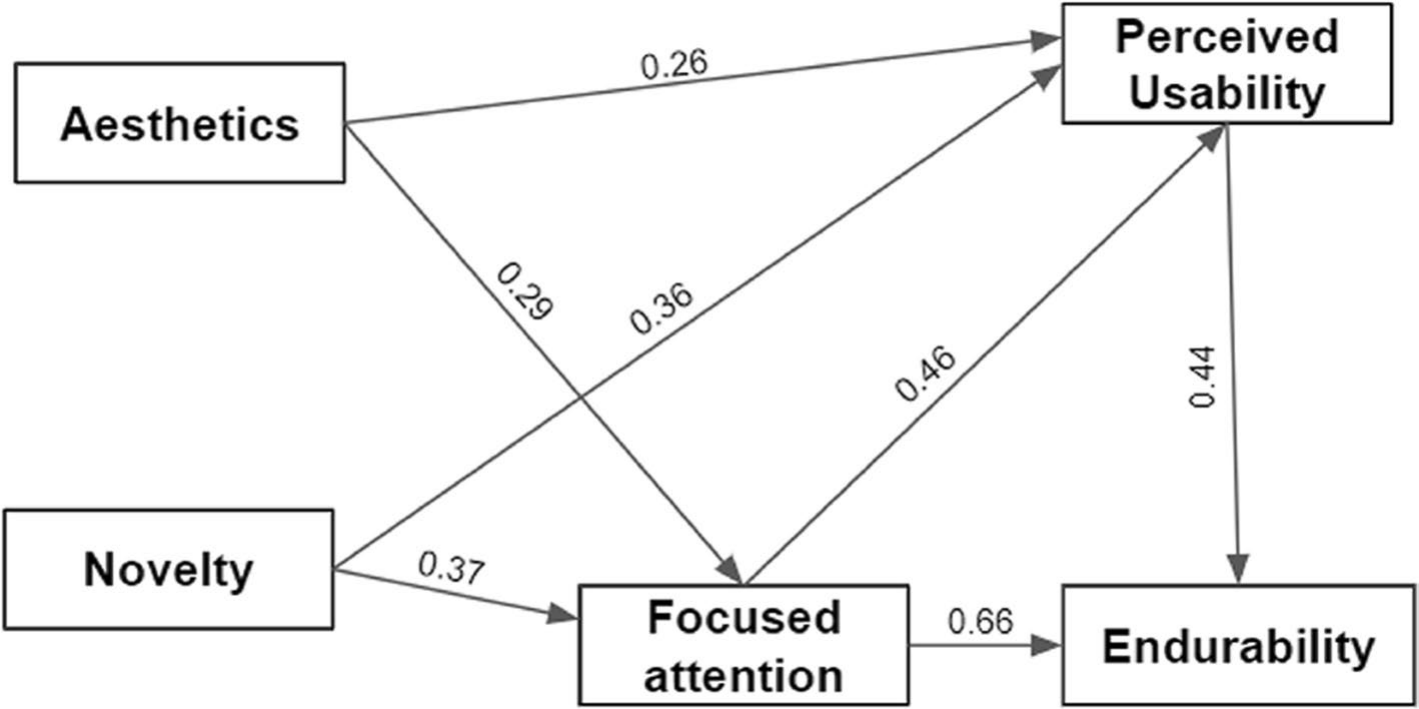
The final structural equation model for the remaining attributes of the UES

**Fig. 3:**
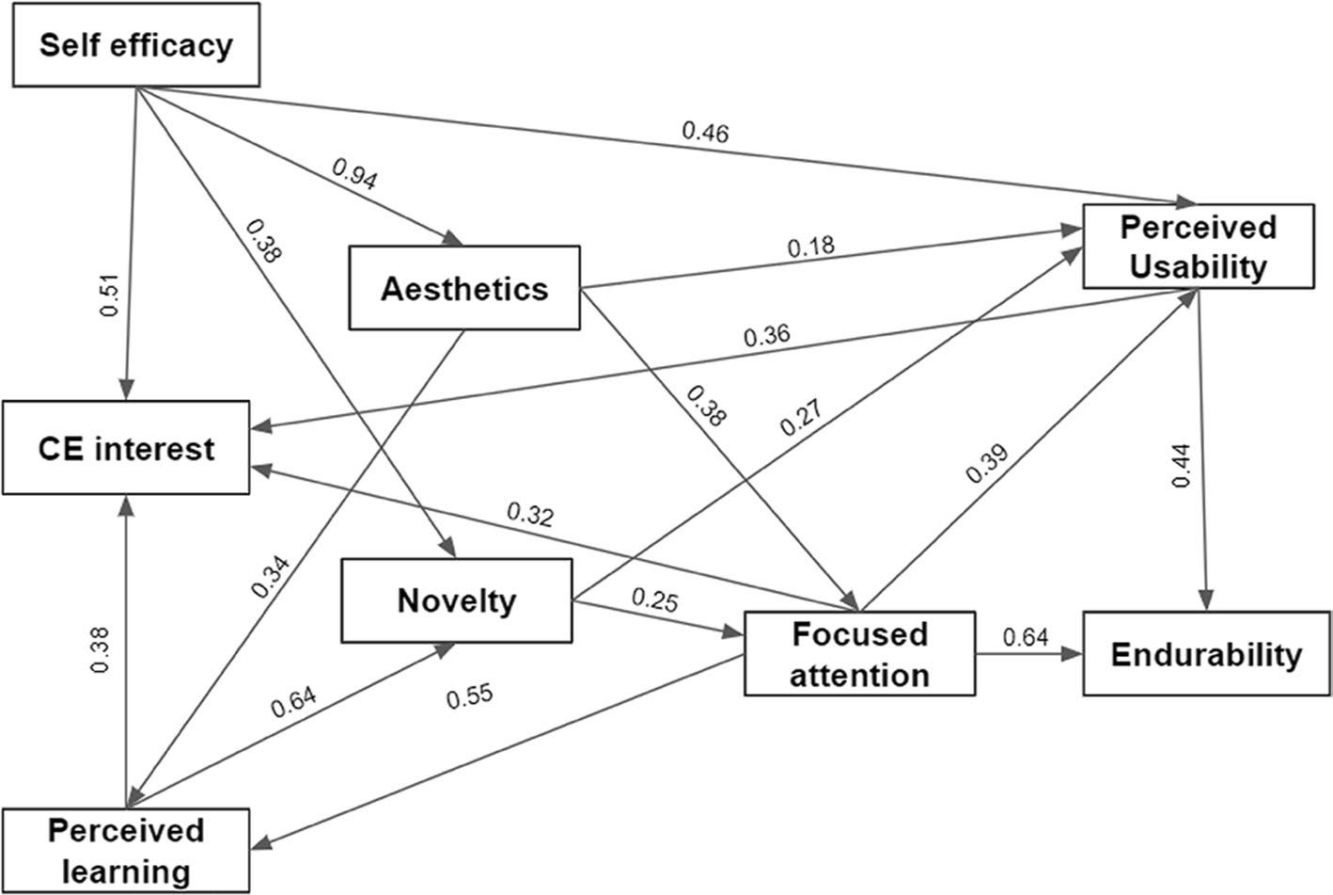
The final structural equation model for eight out of nine attributes with standardized regression weights (significance is < 0.001)

**Fig. 4:**
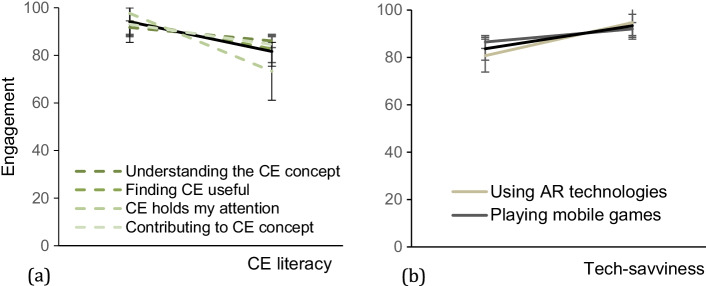
Overall engagement affected by CE literacy shown in the black thick line (a) and tech-savviness (b) (mean agree or disagree statements ± SD, *p* < 0.001). The overall engagement is the result of all attributes assessed via the web survey (self-efficacy, perceived learning, interest in CE, focused attention, novelty, aesthetics, endurability, perceived usability). CE literacy and tech-savviness are defined by factors assessed via the web survey (see legend)

The AR mobile application (CirculAR) used in this study aims to improve citizen awareness and engagement towards CE principles. The app was developed using ARCore and Mapbox for the Unity game engine. RestAPI was used for server communication. CirculAR is compatible with Android smartphones and requires Global Positioning System (GPS) tracking as it supports both marker and location-based applications to overlay the digital data. Marker-based experiences are enabled after scanning a unique QR code for the digital media. The digital data consist of single image and video reveal, image gallery and multiple videos, and static three-dimensional (3D) models. More information regarding the system design and architecture of the AR application can be found in Katika et al. [35]. Figure 1 shows four user interfaces (UIs) of the AR app (Figs. 2, 3, 4).

The user of CirculAR can utilize finger gestures for object manipulation (both rotation and positioning) on the touch-based display of their smartphone [36]. A virtual assistant guides the user; gamification and learning elements ensure a captivating and fun yet educative and engaging experience [37]. The gamification elements include, among others, a scoring system, badges, and a leaderboard. Surveys and quiz questions embedded in the AR content challenge the user’s understanding and provide feedback to enhance the process’s learning effectiveness [38].

The digital media were positioned via a web-based content management tool [35]. This tool allows the selection of the coordinates of each element’s location, the type, content of the media, and the feedback provided and differentiate between the marker and location-activated experiences. The AR experiences’ content was selected to cover a range of difficulty levels. The CE-related content addresses both novice practitioners aiming to understand CE’s basic and core processes and experts aiming to test their knowledge and comprehension and enhance it with additional facts and dimensions [2]. In Fig. 1b, we demonstrate an example of 3D content regarding sustainable tree planting, and in Fig. 1c a quiz question regarding the benefits of composting that the survey participants were exposed. More examples of CE-related content included waste and pollution elimination activities, suggestions for product and material re-use, and regeneration of natural systems [10]. In total, the participants were exposed to 20 elements of the CE concept and methodology through AR content.

### Web Survey

For the period that the present study was conducted, in situ tests were not allowed due to the COVID-19 restrictions in the country. Therefore, virtual material and consultation, as well as online distribution were the sole means to expose participants to our AR engagement tool. To assess the information required regarding the attributes and factors affecting engagement, and to inform users about the AR engagement tool, we conducted an extended web survey, which consisted of three parts:

- The first part aimed to assess the factors affecting engagement. More specifically, the technological profile (use of a smartphone, AR features, camera, and mobile games) and the users’ demographic data were collected. In this part, ten questions aimed to assess the participant’s overall understanding and confidence towards the CE principles.
- The second part of the survey included a video demonstrating the AR application’s use, features, and content. The overall video lasted 3 min, and the users were unable to proceed to the third part of the survey unless having watched the video. This part exposed the users to the media and functionalities of the AR engagement tool.
- In the third part of the survey, changes in the user’s perceived comprehension of the CE principles and confidence towards such an economic approach upon exposure to the AR engagement tool were assessed. At the same part of the survey, the nine attributes affecting engagement were also assessed.

To ensure the UES’s robustness and that the attributes adequately capture our proposed construct, we prepared a questionnaire as part of the third part of the web survey, based on relevant work by O’Brien and Toms [32]. Using this approach, we ensured the required variability in the scale scores without limiting the potential to detect differences between samples, experimental conditions, and systems. A few items have been modified to meet the purpose and structure of the AR engagement tool since the initial survey was prepared in the context of online shopping and marketing. Attributes associated with the motivational engagement were also added to the third part of the web survey based on previous studies by McLean and Wilson [28]. Finally, factors that assessed the participants’ tech-savviness and CE literacy and confidence and general demographics were also included in the questionnaire. These factors aimed to reveal information regarding the inclusivity of the tool and guided the answers to the research questions.

### Recruitment of Citizens of a Municipality

The web survey was distributed to the citizens of Karditsa, Greece. Karditsa is an evolving peripheral town that belongs to the Municipality of Karditsa, Region of Thessaly — Central Greece. The survey was translated to the native language of the citizens (Greek) to avoid any language barriers or bias. The city of Karditsa was selected primarily due to the sectors, related objectives, and priority axes set in the public space strategic management plan [39]. The city aims to establish citizens’ interaction and cooperation, promote the CE model, adopt and use ICT tools, reduce their energy footprint, adopt participatory processes in local decision-making and good practices, cultivate environmental consciousness, and integrate new green spaces and sustainable urban mobility. Therefore, the use of an AR application to improve Karditsa’s citizens’ engagement in CE is very relevant.

To ensure proper web survey methodology, we followed the core web survey process proposed by Callegaro et al. [40]. The process suggests three distinct steps: pre-fielding, then fielding, and finally, post-fielding activities. During pre-fielding, we set the sample size and sample design and designed the questionnaire. The sample size was defined according to the ratio of sample size to the number of free parameters. We estimated approximately 180 participants (for the nine attributes of engagement) for a successful study (ratio 20 to 1 according to [41]. We established the recruitment process, and the citizens were recruited via local blogs and forums during March 20–31, 2021. We did a soft launch of the survey with a subset of respondents during March 15–20, 2021. This soft launch offered the opportunity to conduct first quality checks and detect technical problems. The analysis discarded the first 20 responses that provided such information and feedback. Minor revisions in the formulation of the questions were performed upon the feedback of the initial participants to ensure clarity. Upon fielding, we continued with the data preparation. The outcomes of the analysis, processing, and valorization of the results are presented in this paper’s “Results” section.

### Recruitment of General Population

To support the model suggested to the municipality, we distributed our web survey through open channels to address citizens outside of the municipality, as part of a general population. The survey was distributed in English. To ensure that many interested stakeholders participated in our study, we recruited users via groups related to AR and CE on popular social platforms, such as Facebook and LinkedIn. Thus, this was a purposive sample with individuals chosen based on their interaction with the applications of interest to this study. The survey was posted online for 2 weeks (March 15–31, 2021). For the first 5 days, we collected feedback from 20 participants to optimize the survey and correct any technical issues. These responses were discarded by the analysis.

### Measures

The questions in the web survey assessing the engagement and factors affecting engagement used the Likert scale to demonstrate the agreement of the participant with statements. We used both positive and negative frames in the statements to ensure the participants are not biased towards agreeing with all of them to show themselves in a positive light and that the participants’ responses are reliable and consistent. The questionnaire consisted of 67 items that requested participants to indicate their agreement level, from “strongly agree” to “strongly disagree” (with intervals from 1 to 4 accordingly).

We chose four maturity levels for the Likert scale to enable the user to form a clear opinion on each statement. As we made sure that all questions apply to our AR application user, we concluded a specific user opinion is essential. We chose a unipolar scale to measure the attribute of agreement in each statement that was treated as interval level. Overall, we used descriptive statistics to summarize the data we collected, but we also analyzed single Likert-type questions for deeper insights into specific attributes presented in the discussion. Since we kept participation in the survey relatively high and acquired an adequate sample size where the data are normally distributed, parametric tests were used with Likert scale ordinal data [42]. Since there were no missing responses and no patterns of incoherent answers were observed, no questions were excluded from further analysis.

## Results

### Participants

In total, 127 valid responses were collected by the web survey distributed to the citizens of Karditsa, Greece. All participants of the municipality viewed the demo of the AR application and answered all the mandatory questions in the survey. In parallel, 101 valid responses were collected via the web survey addressed to the general population outside the municipality. Table 1, summarizes the participant demographics and their exposure to technology through the use of smartphones and AR technologies.

**Table 1:** Participant demographics

	*N*_Karditsa_	%	*N*_global_	%
Sex				
Male	44	34.6	52	51.5
Female	80	63	42	41.6
Other	3	2.4	7	6.9
Age				
18–25	12	9.4	5	5
26–35	39	30.7	55	54.5
36–45	19	15	32	31.7
46–55	37	29.1	6	5.9
56–65	19	15	3	3
Education				
School	11	8.7	0	0
Bachelor	86	67.7	17	16.8
Masters	25	19.7	54	53.5
PhD	5	3.9	29	28.7
Other	0	0	1	1
Tech-savviness				
Mobile gaming	84	66	60	60
Use of AR	73	57.5	78	77
Location				
Karditsa	127	100	0	0
Greece			38	38
Italy			6	6
The Netherlands			10	10
Denmark			18	18
Belgium			5	5
Sweden			3	3
Switzerland			4	4
UK			8	3
Hungary			6	6
Other			3	3
Total participants	127		101	

### Preliminary Analysis

The low participation in the web survey, outside of the municipality of Karditsa, did not qualify for a reliable analysis of the data extracted. Trends in the factors will be extracted in the following sections to demonstrate any correlation with our case study within the municipality (city of Karditsa) outcomes. Therefore, all paragraphs therein refer to the engagement attributes assessed by the survey contacted with the citizens of Karditsa.

The User Engagement Scale’s reliability and validity of the survey has been evaluated with six attributes (perceived usability, aesthetics, novelty, felt involvement, focused attention, and endurability) applied in online shopping [24]. To ensure that the UES is reliable in the context of online engagement tools too, we performed once more the confirmatory factor analysis and determined Cronbach’s alpha. The results of this initial stage of analysis confirmed that the scale was multidimensional and that the items loading on each attribute were consistent with the findings of the previous study [24].

#### Factor Analysis

Using IBM SPSS Statistics 21, we aimed to understand whether the attributes of the User Engagement Scale load together to capture the user’s engagement. A similar approach was followed for the attributes of motivational engagement. Felt involvement was removed by the hypothesized model since the a-determinant was significantly lower due to this attribute. Our preliminary assumption is that users are not able to experience felt involvement of a mobile app through a video.

For all remaining attributes of engagement assessed via the web survey in the municipality, we determined Cronbach’s alpha as a measure of internal consistency reflecting how closely related a set of items are as a group. Values of the parameter from 0.7 to 0.9 were considered “respectable” to “very good,” and values above 0.9 were considered “excellent” [43]. Table 2, shows the alpha values for all the engagement attributes.

**Table 2:** Reliability estimates of factors

Factor	Nr items	Cronbach’s alpha
Aesthetic appeal	3	0.93
Perceived usability	3	0.87
Novelty	3	0.90
Endurability	3	0.84
Focused attention	2	0.84
Interest in the topic	4	0.88
Perceived learning	3	0.88
Self-efficacy	2	0.93

Principal component analysis (PCA) was then run with all items to ensure that a single factor did not emerge. Correlations for the measures used in the study were calculated (Table 3). Upon removing felt involvement, all remaining attributes appeared to be associated with each other, and all correlations were significant at the 0.01 level [24]. The correlation between all attributes had associations between 0.6 and 0.9. Overall, there was internal consistency among the six attributes. The results of this initial stage of analysis confirmed that the scale was multidimensional and that the items loading on each attribute were consistent with the findings of the previous studies by O’Brien and Toms. After that, we were able to proceed to the structural equation modeling (SEM) analysis confidently.

**Table 3:** Intercorrelations for factors

	Self-efficacy	Perceived learning	Novelty	Aesthetic appeal	Focused attention	Perceived usability	Interest in the topic	Endurability
Self-efficacy	1							
Perceived learning	0.745	1						
Novelty	0.639	0.789	1					
Aesthetic appeal	0.606	0.695	0.614	1				
Focused attention	0.616	0.793	0.75	0.705	1			
Perceived usability	0.744	0.783	0.783	0.719	0.793	1		
Interest in the topic	0.782	0.829	0.769	0.639	0.782	0.833	1	
Endurability	0.689	0.815	0.841	0.701	0.874	0.867	0.817	1

### Structural Equation Modeling (SEM)

#### Structural Equation Model for UES

According to Wolf et al. [44], a minimum of 40 participants per attribute is required for satisfactory analysis. Even though our sample within the municipality was less than 320 participants (for the eight attributes of user engagement) to ensure the validity of structural equation modeling, we pursued this step to demonstrate conformance to the initial study of O’Brien and Toms [24]. Our first step was to verify the final structural equation model proposed, given the removal of one of the attributes (felt involvement).

The fitness of the questionnaire’s internal structure was evaluated with confirmatory factor analysis, employing SPSS AMOS 26. The model suggested that endurability was predicted by perceived usability and focused attention. Aesthetics predicted perceived usability and focused attention; novelty predicted focused attention and perceived usability; and the structural model becomes as shown in Figure 2.

#### Structural Equation Model for All Attributes Predicting Engagement

Following the CFA’s goodness of fit and satisfying the subsequent tests, the structural equation model was then estimated. We performed multiple analyses, and we excluded all values below 0.2. The final structural equation model is presented in Figure 3. The predicted relationships with their regression (*β*) and significance (*p*) are presented in Table 4.

**Table 4:** SEM regression estimates (***: not significant)

			**Regression**	**Significance**
Aesthetic appeal	←	Self-efficacy	0.936	***
Novelty	←	Self-efficacy	0.376	***
Focused attention	←	Aesthetics	0.376	***
Perceived learning	←	Aesthetics	0.341	***
Perceived usability	←	Self-efficacy	0.456	***
Perceived usability	←	Focused attention	0.386	***
Perceived usability	←	Aesthetics	0.181	0.006
Perceived usability	←	Novelty	0.275	***
Interest in the topic	←	Self-efficacy	0.467	***
Interest in the topic	←	Perceived usability	0.475	***
Interest in the topic	←	Perceived learning	0.511	***
Endurability	←	Focused attention	0.641	***
Endurability	←	Perceived usability	0.438	***
Novelty	←	Perceived learning	0.645	***
Focused attention	←	Novelty	0.25	***
Perceived learning	←	Focused attention	0.548	***

In each case, the following indices of fit were calculated: root mean square error of approximation (RMSEA), goodness of fit (GFI), normed fit (NFI), and comparative fit (CFI). The proposed values for excellent fit are described in Hooper et al. [45] as the following: CFI, NFI, GFI, TLI > 0.95 and RMSEA < 0.08. Kelloway [46] suggests that higher values of RMSEA reaching 0.1 also suggest a good fit.

As expected, given the low number of participants, the resulting model did not have an ideal fit, but since our results are in close agreement, we proceeded with further analysis. For the 127 participants, we determined *χ*^2^ = 51, df = 11, *p* < 0.01, CFI = 0.938, NFI = 0.923, GFI = 0.947, TLI = 0.945, and RMSEA = 0.103.

### User Engagement Attributes

To perform descriptive statistics with the Likert scale results, we assumed the difference between each point in the scale to be evenly spaced [42]. Table 5 shows descriptive statistics for the eight attributes assessed to describe engagement. For all attributes, the mean scores vary from 1.4 to 1.8, and the variance is below 0.7.

**Table 5:** Descriptive statistics for the eight attributes (excl. felt involvement) used to describe engagement

		Mean	SD	Variance
Self-efficacy	Using the app would help me understand the basic concepts of CE	1.59	0.59	0.34
	Using the app would help me explain basic concepts of CE to a friend	1.60	0.64	0.40
Perceived learning	The learning activities in the AR app appear meaningful	1.46	0.61	0.37
	The AR app would stimulate my curiosity to learn new things	1.53	0.69	0.47
	Using the app would help me learn factual information about CE	1.50	0.58	0.33
Interest in the topic	The AR app stimulates my interest in CE	1.72	0.67	0.44
	Using the app would help me engage in Circular Economy principles	1.73	0.67	0.44
	Using the app would help me feel interested in CE	1.65	0.81	0.66
	I feel positive to use the AR app	1.70	0.65	0.41
Focused attention	Spending time on AR seems worthwhile	1.69	0.79	0.62
	Learning through AR would help me focus more on CE	1.71	0.70	0.49
Aesthetics	The AR app seems attractive	1.56	0.61	0.37
	The AR app appears aesthetically appealing	1.55	0.64	0.41
	I liked the graphics and images in the AR app	1.54	0.61	0.37
Endurability	My experience would seem rewarding	1.59	0.67	0.45
	I would recommend the AR app to my friends and family	1.69	0.74	0.54
	Learning about Circular Economy through AR seems worthwhile	1.68	0.68	0.46
Novelty	I would use the AR app out of curiosity	1.67	0.76	0.57
	The content of the AR app incited my curiosity	1.60	0.74	0.55
	I feel interested in the AR application	1.63	0.69	0.48
Perceived usability	I feel excited to use the AR app	1.76	0.76	0.57
	I would be satisfied with this type of the activity	1.66	0.69	0.47
	The AR experience doesn’t seem demanding	1.74	0.74	0.54

### Factors Affecting the Engagement Attributes

In our survey, we extracted several factors to investigate their effect on the engagement attributes. These factors included the CE literacy, tech-savviness, and demographics of the 127 participants from the citizens of the municipality of Karditsa.

Starting with the participants’ exposure to technologies, such as smartphones, AR features, mobile games, cameras, and search engines, we established that more than 90% of the participants use a smartphone, perform web searches, and use their smartphone to take photos. Therefore, only exposure to AR and mobile games should determine how tech-savviness affects engagement attributes. As for the CE literacy, the variation in all assessed factors allowed us to proceed with the correlation analysis. Figure 4 (a and b) demonstrates how all engagement attributes are affected by tech-savviness and CE literacy.

**Fig. 5:**
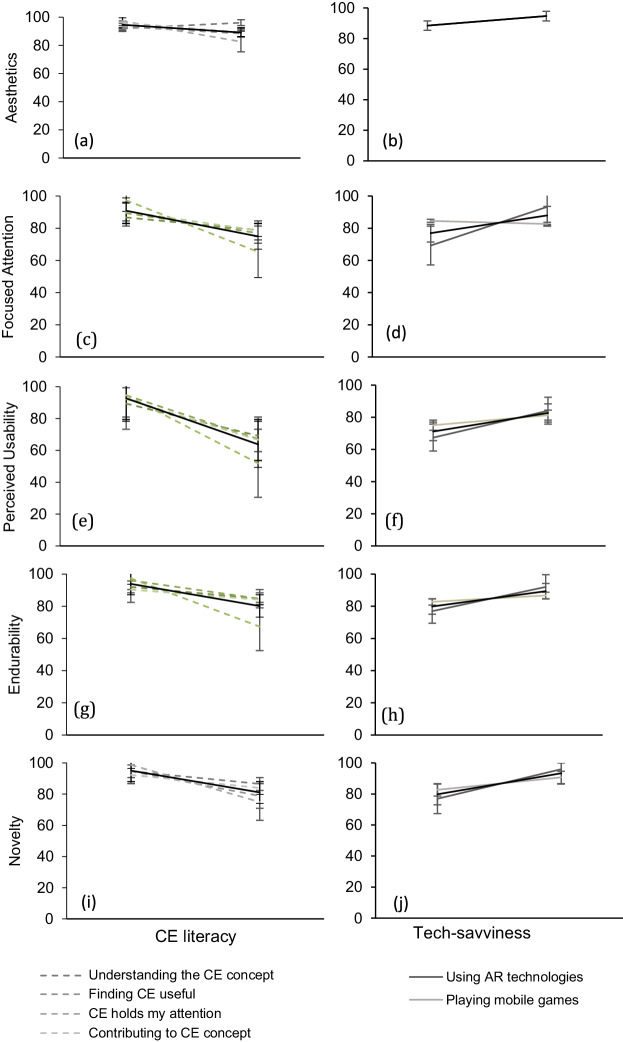

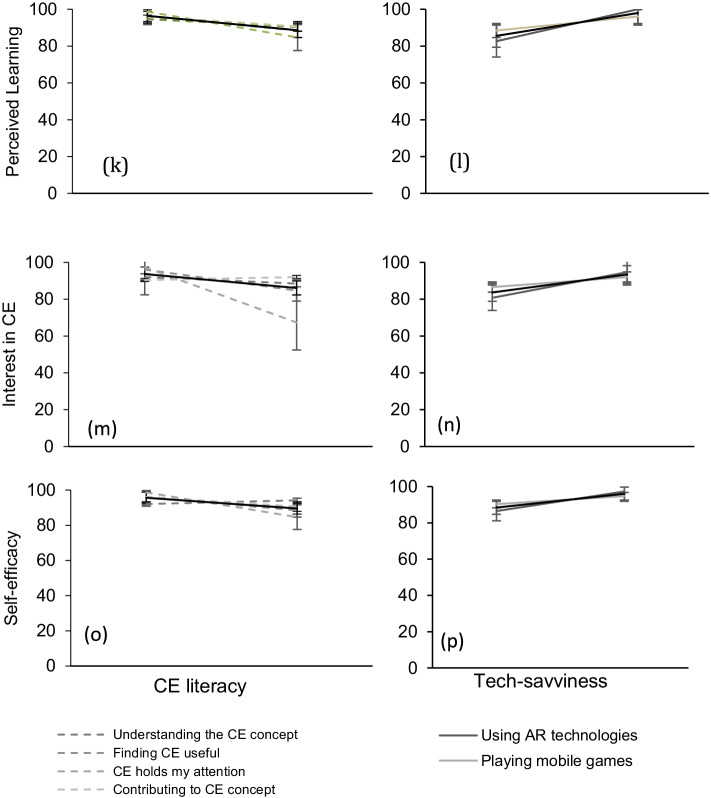
The effects of CE literacy (first column) and tech-savviness (third column) on eight engagement attributes (mean agree or disagree statements ± SD, *p* < 0.001). The CE literacy is the result of four factors assessed via the web survey (demonstrated in the legend of the graph). The tech-savviness is the outcome of the use of AR and mobile games from all 127 participants of the municipality. The black thick lines in all graphs describe the average of the factors affecting each attribute

**Fig. 6:**
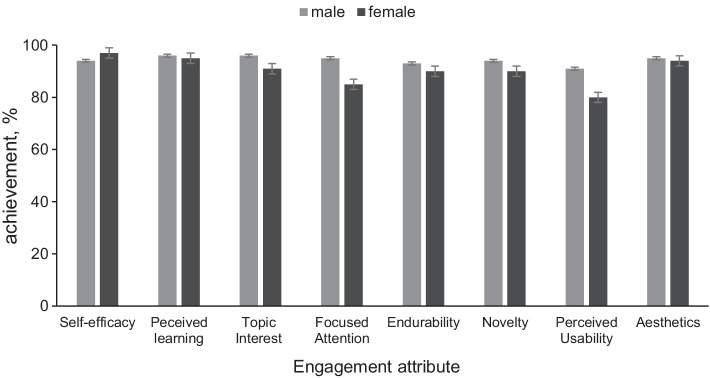
Gender effects on the engagement attributes (mean agree or disagree statements ± SD, *p* < 0.001). No significant observations can be made regarding the differences in the engagement among the genders participating in the study

**Fig. 7:**
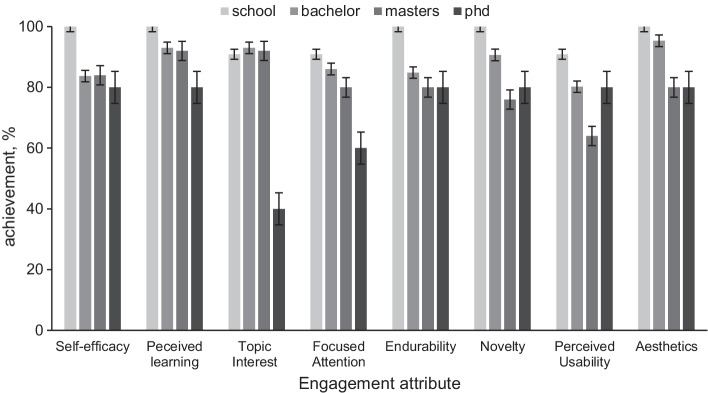
Education of the participants affecting the engagement attributes (mean agree or disagree statements ± SD, *p* < 0.001). Most participants without a higher education degree scored higher in most engagement attributes

**Fig. 8:**
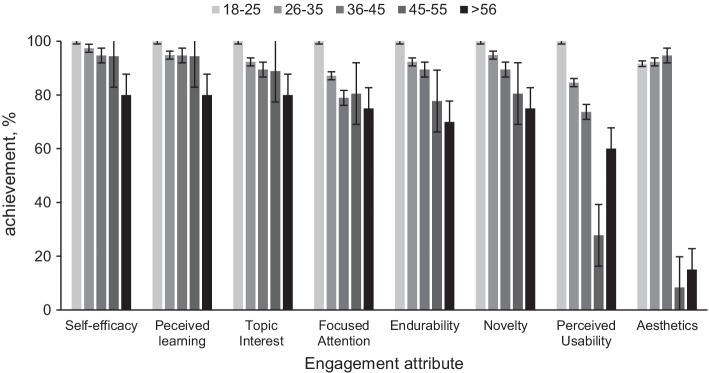
Age of the participants affecting the engagement attributes (mean agree or disagree statements ± SD, *p* < 0.001). Participants older than 45 years fail to see the perceived usability and aesthetics of the app as demonstrated by the last two attributes compared to younger participants

Figure 5 goes into more details and demonstrates how individual factors affect each engagement attribute separately. The factors have been summarized in two main categories; CE literacy and tech-savviness (the individual factors are shown in the legend of the figure). Increase in the CE literacy of the end-user of the tool results in a reduction in the overall engagement assessed by all attributes. On the other hand, the more tech-savvy is the participant, the highest the engagement. Users exposed to AR understood the usability of the AR app 25% more, compared to users who were seeing AR technology for the first time. Overall, exposure to AR is a factor affecting most engagement attributes from 10 to 25% as seen in the graphs of the second column in Fig. 5. Such a finding contributes to the perception that tech-savviness expressed by the exposure to mobile games and AR may affect citizen engagement via this tool (Figs. 6, 7, 8).

While investigating the engagement attributes separately, one of the factors that demonstrated the highest variance comes from end-users who claimed that CE already holds their attention before exposure to AR, most likely, demonstrating highest CE literacy and confidence. These users resulted in 10–15% lower in all engagement attributes (Figure 4a). They also resulted in 30% lower interest in CE upon exposure to the AR engagement tool (Fig. 5 — first column). The same users failed to see the mobile app’s usability to a degree almost at 50% (Figure 5e). Their endurability was also lower to a degree of 30% (Figure 5g). All these observations contribute to the assumption that the AR app is more engaging to participants that have not shown any prior interest in CE principles.

On the same note, users who were already confident to practice the CE practices or to explain the CE principles to their friends appear to miss out on the AR app’s perceived usability to a degree of 20 to 30% (Fig. 5e). Participants who already understood the CE principles and were confident that CE is a useful concept failed to see the AR app’s usability to a degree of more than 30% compared to users who do not have the same understanding and perception of use before the exposure to the AR app. The effect of CE literacy on the perceived usability is shown in Figure 5e and f.

The same trend is followed by another factor; users who already wanted to contribute to CE found the AR engagement tool almost 30% less useful than users who had not expressed their interest to contribute to CE (Figure 5e). Confident users paid 12% less attention to the AR app. All findings contribute to the outcome that participants who already had high levels of literacy towards the CE topic before participating in our study demonstrate a significant decrease in their engagement.

Despite the difference in the participation between males and females, no significant differences are seen in all engagement attributes (Figure 6). Differentiations of less than 10% may hint at a finding but require further investigation to extract a concrete outcome (Table 1). The familiarity with CE principles and methodology appears to correlate with neither age nor gender. A tendency towards higher engagement of participants who do not have higher education is observed as they consistently score higher than other participants in most attributes (Figure 7). The results suggest they tend to feel more confident towards CE after the exposure to the AR app and are more likely to recommend such an experience to their friends.

Age appears to affect many engagement attributes as seen in Figure 8. Participants of higher age appear to disagree with the aesthetics and perceived usability of the AR app. We proceeded by analyzing other determining factors of such a response. We realized that similar percentages of males and females exist in all age groups (50–70% females and 30–50% males); therefore, gender had no impact. Similarly, in all age groups, 5–20% of the participants do not have a university degree, so education is not a determining factor. On average 50% of the users have never been exposed to AR technology without notable fluctuations in each age group. The only factor that stood out was that almost 60% of the participants older than 46 do not play mobile games, contrary to approximately 20% of the participants aging below 45. Even though participants who play mobile games do not demonstrate high discrepancies in the engagement attributes, it appears that the combination of these two factors is a determining factor for the perceived aesthetics and usability.

### Contribution from the Global Survey.

**Fig. 9:**
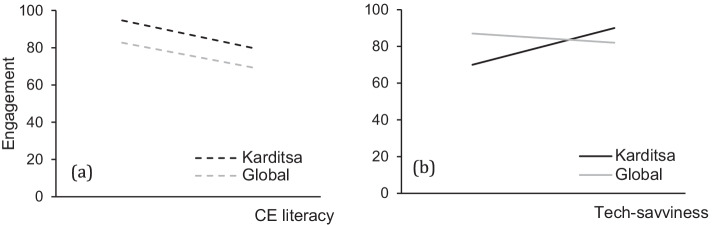
Overall engagement affected by CE literacy (a) and tech-savviness (b). The CE literacy is assessed by participant’s willingness contributing to CE, understanding of the CE and its usefulness, and participants who claimed that CE holds their exposure. The tech-savviness is assessed by the exposure of the participants to AR technology. All factors assessed before exposure to the AR engagement tool (mean agree or disagree statements ± SD, *p* < 0.001). The overall engagement is the result of all attributes assessed via the web survey (self-efficacy, perceived learning, interest in CE, focused attention, novelty, aesthetics, endurability, and perceived usability)

To investigate the validity of our questions and extract preliminary results to an extent more significant than that of the municipality in Greece, we performed a similar analysis with the independent sample that answered the global survey. We collected most answers from the capitals of Greece, Denmark, and the Netherlands. Even though the response rate was low, we proceeded by investigating the most determining factors of the overall engagement from the previous analysis, the CE literacy and tech-savviness. Similar trends are observed regarding the CE literacy in both surveys and validated our initial results. CE-proficient participants scored lower in all engagement attributes. Figure 9a is showing how CE literacy before exposure to AR is affecting all engagement attributes both from the survey distributed to the citizens of Karditsa and globally. On the contrary, tech-savviness did not follow the same trend in the global sample as in the municipality one. Figure 9b demonstrates the effect of the tech-savviness of the participants on the engagement attributes and enhanced tech-savviness appears to compromise engagement. The absence of participants above the age of 45 in the global survey may have affected the outcome.

## Discussion

Guided by the research questions, our study suggests that AR, acting as an engagement tool, can increase end-users’ perceived interest and engagement in CE concepts. At the same time, it can help narrow the gap between public authorities and citizens, which has been found to impact CE adoption [47, 48]. Results reported here support findings by other researchers who argued that AR could help change one’s perspective or frame of reference, allowing them to understand complex phenomena such as novel economic models and concepts [49]. They also support research findings investigating the positive impact of ICT technologies on citizen engagement [8, 9]. Finally, addressing the significant limitations of current ICT engagement practices, our results indicate increased social inclusion and community cohesion, fast technology diffusion, and relevance [4, 6, 8].

Answering the first research question, one of this study’s main findings is that our AR tool had a more pronounced effect on the engagement of participants who have low CE literacy on the topic. It should be noted that general public exposure to CE principles is relatively low and new, providing confidence in our tool’s relevance [3]. The ability to influence citizens with low CE literacy is of high importance as it demonstrates this tool’s ability to foster citizen engagement in matters that have not reached a maturity of understanding which is a prerequisite for broad adoption. Such an approach adds to the list of ICT tools that may contribute to citizen engagement promoting transparency, participation, and collaboration between government and the citizenry [4, 9, 50].

A very promising finding, revealing the relevance of the second research question, is that the gender of the end-users is not affecting the engagement and interest in the topic we convey via AR. A thorough investigation on the motivational factors regarding the climate change mitigation performed by Brink and Wamsler [51] demonstrated that females are more inspired by social matters while similar inspirations among both genders were observed for technical issues. Since the present research focused mainly on assessing technological attributes affecting engagement, similarities among the genders participating in the study were expected.

Upon exposure to the AR engagement tool, more than 75% of all users in this survey reported a high interest in CE, and more than 83% of all participants demonstrated high self-efficacy and perceived learning in the same topic. The results are higher for users who do not have higher education, agreeing with Billinghurst and Duenser [17] who demonstrated that AR might enhance the traditional learning models as interactivity seems central to content engagement. Similarly, Makransky et al. [52] reported that end-users with lower literacy levels on a specific topic benefit more from immersive techniques, especially when combined with traditional learning establishments. Thus, compared to other citizen engagement initiatives based on web-based platforms that demonstrated a significant increase in participation among citizens of higher education, the AR engagement tool is more inclusive towards the population with a lower education level [8].

To further support the AR engagement practice’s inclusivity and answer the third research question, the citizens of the municipality demonstrated high self-efficacy regardless of any exposure to similar technology. Self-efficacy is a significant predictor of success associated with the ability to complete tasks, and was not affected by the low prior exposure to AR [30]. Nearly all participants reported that they were motivated and more confident to practice CE principles, while almost half of them had never been exposed to this technology. Overall, our findings suggest that an AR app is valuable to teach complex concepts and empower competencies that are hard to attain, especially for people who do not have access to higher education.

The citizens of Karditsa have articulated their need to follow a more circular approach by expressing their dissatisfaction due to the lack of effective waste management processes in the town center and with their access to information that affects public matters [39]. They have also disclosed their interest in increasing their environmental awareness and effective waste management while also expressing a positive stance to engagement through digital participation means [39]. Our findings come to an agreement with the citizen’s beliefs since more than three-fourths of all participants felt interested in the AR-specific CE-related context, which emphasized the need for change towards a more circular and sustainable future. The increased relevance and value of solutions proposed and later generated through the engagement process contribute to increased community cohesion [4].

Younger age groups in the municipality of Karditsa demonstrated high dissatisfaction in terms of environmental issues, which appear to be their preferences on prioritization of local resource distribution for meeting citizens’ expectations [39]. Such a factor could explain the significant increase in the engagement attributes for participants aged less than 45. The improved engagement was anticipated since a citizen engagement tool is more successful when it considers the participants’ input and demonstrates an understanding of needs, problems, and priorities. At the same time, the success of an engagement process is dependent on the ability to acquire local knowledge and experience and consider the citizen’s aspirations and values [4]. More research is required to define whether the increase in engagement among younger participants reflects their established dissatisfaction with current city initiatives or their highest exposure to mobile games.

Upon exposure to our AR engagement tool, the citizens of Karditsa agreed that an AR app would positively affect their understanding of CE principles and their ability to apply them in real life to a degree of 88%. Such a promise is fundamental to improve the use of the city resources since the citizens of Karditsa are not committed to an inclusive, sustainable, city- and citizen-specific, strategic public space management and regeneration plan, and their perception of it as a “common good” is relatively poor [39]. There is a lack of open governance regarding public space management since the local community has no role in relevant decision-making processes.

### Future Studies and Limitations

This study investigated whether an AR app can increase citizen engagement in CE-related topics. Due to AR’s infancy as an engagement tool, a limited number of apps utilize AR to its fullest potential. Additionally, since not many societies have adopted CE, there is a limited number of interested (or well-informed) users. Given the promising nature of our results, in terms of inclusivity, relevance, accessibility, and educative means, other municipalities can use the example of the city of Karditsa to improve their citizen participatory practices. The municipality is planning an in situ activity upon seizing the COVID-19 restrictions to investigate the impact of this initiative on the citizens. The AR engagement tool will be distributed to the citizens, and surveys and questionnaires will assess the before and after changes in their behavior and mindset as an effect of the actionable and timely information available on their mobile devices. The citizens will report changes in performance and other initiatives to reflect whether the AR engagement tool contributed to more sustainable lifestyle choices.

The inability to correlate felt involvement with engagement (as described in the preliminary analysis), despite suggestions from other research studies that claimed to be one of the determining attributes of engagement, reflects the need for future research to explore this attribute as well as assess its effectiveness. Future research should expose the participants directly to the AR engagement tool via in situ tests (as soon as COVID-19 restrictions allow it) to ensure better insights into such an attribute. An experiment-based research design may allow researchers to gain further insight into the unique AR attributes influencing citizen engagement towards CE adoption.

In the case studies reported in this paper, we examined how age, gender, tech-savviness, prior exposure to related content, and education level affect such an adoption. A future study should consider additional vital dimensions such as disabilities, income, cultural or language differences, and geographical or social isolation of the participants.

Lastly, a limitation of this work lies in its use of an online survey as a data collection method. To ensure that the participants had a great understanding of the AR app, they watched a lengthy video demonstrating the AR app’s content, features, and capabilities. Plus, to assess all user engagement attributes and all factors affecting these attributes, the users had to respond to 44 questions. Therefore, our response rate was low.

## Conclusions

In this work, we reported on the development of a novel, inclusive, accessible, and portable citizen engagement tool that utilizes AR technology. We assessed its ability to improve the attributes that the literature suggests to predict engagement, along with the factors that affect these attributes. The research was conducted both in a closed urban environment, the municipality of Karditsa, and globally.

The study demonstrated significant improvement in context-specific citizen engagement. Specifically, both the municipality citizens and a global sample of participants demonstrated high motivation towards CE and an enhanced self-efficacy in the same topic. Overall, all attributes demonstrated a good correlation with end-user engagement.

With regard to inclusivity requirements, the tool was found to be more useful to citizens with low CE literacy and confidence than to CE-proficient participants. The tool’s usability also increased with the dissatisfaction with the current city initiatives towards a more sustainable environment. Upon exposure to the AR engagement tool, citizens who did not have higher education feel more confident towards CE and are more likely to recommend the experience to their friends. Such an outcome points towards the ability of AR-driven engagement to make information that affects public matters accessible to everyone.

We conclude that in communities aiming to change their participatory approaches and governance schemes towards climatic and economic issues, such an AR engagement tool supports sustainable change and adoption, providing an inclusive and democratic engagement platform appropriate for citizens of all ages, education levels, tech-savviness, and genders (Tables 1, 2, 3, 4, 5).

## Supplementary Information

Below is the link to the electronic supplementary material.

Supplementary file1 (DOCX 17 KB)

## Data Availability

The authors confirm that the data supporting the findings of this study are available within the article and Supplementary material.

## Code Availability

Code availability is not applicable to this article as no new code were created or analyzed in this study.
