# Supplementary material for: Augmented Reality (AR) Supporting Citizen Engagement in Circular Economy
Source: Circ Econ Sustain. 2022 Feb 3;2(3):1077–104. doi: 10.1007/s43615-021-00137-7 (PMC8820368; doi:10.1007/s43615-021-00137-7)
Supplement: Supplementary file 1 — Supplementary file1 (DOCX 17 KB) [file 43615_2021_137_MOESM1_ESM.docx]

**Citizen engagement in Circular Economy principles using an AR engagement tool**

Please indicate your:

1. Age (18-20/21-30/31-40/41-50/51-60/61-70/71-80)
2. Gender (male/female/other)
3. Education level (School/Bachelor/Masters/PhD/Other (please specify))
4. Location (name of the City/Country)

Please choose the most appropriate response from the statements below, to each question before using the AR app:

Statements: *Every day - Few times per week - Few times per month - Never*

**Tech-savviness**

1. Ι use a smartphone device
2. I play mobile games
3. I use Augmented Reality (AR) technology (such as, camera filters, mobile apps)
4. I use a smartphone to learn new things (ie. Google or Wikipedia)
5. I use a phone for regular things (such as, calling and texting)
6. I use email and perform web-searches

**CE-literacy**

1. I don’t understand the concept of Circular Economy (CE)
2. I am not sure whether CE is a useful concept
3. I am familiar with the benefits of CE approach
4. I would describe engaging to CE as a fun thing to do
5. CE doesn’t hold my attention at all
6. I want to contribute to the CE water cycle
7. I want to know more about CE but I am unsure how to do it
8. I am confident and can understand the basic concepts of CE
9. I am confident that I can explain concepts of CE to a friend

Please indicate your level of agreement using one of the statements below, by choosing the most appropriate response to each question:

Statements: *Completely disagree - Mostly disagree - Mostly agree -Completely agree*

**Self-efficacy**

1. Using the app would help me understand the basic concepts of CE
2. Using the app would help me explain basic concepts of CE to a friend

**Perceived learning**

1. The learning activities in the AR app appear meaningful
2. The AR app would stimulate my curiosity to learn new things
3. Using the app would help me learn factual information about CE

**Interest in the topic**

1. The AR app stimulates my interest in CE
2. Using the app would help me engage in Circular Economy principles
3. Using the app would help me feel interested in CE
4. I feel positive to use the AR app

**Focused Attention**

1. Spending time on AR seems worthwhile
2. Learning through AR would help me focus more on CE

**Aesthetics**

1. The AR app seems attractive
2. The AR app appears aesthetically appealing
3. I liked the graphics and images in the AR app

**Endurability**

1. My experience would seem rewarding
2. I would recommend the AR app to my friends and family
3. Learning about Circular Economy through AR seems worthwhile

**Novelty**

1. I would use the AR app out of curiosity
2. The content of the AR app incited my curiosity
3. I feel interested in the AR application

**Perceived Usability**

1. I feel excited to use the AR app
2. I would be satisfied with this type of the activity
3. The AR experience doesn't seem demanding

**Felt involvement**

1. I would be drawn into the virtual content of AR
2. The AR experience seems fun
